# Evaluating the CASA model for estimating carbon sequestration in sea buckthorn plantations using multi-temporal remote sensing and field data

**DOI:** 10.48130/forres-0026-0013

**Published:** 2026-04-09

**Authors:** Maoyuan Wang, Yanrui Guo, Shaodong Liu, Peng Zhang, Yan Gao, Shi Qi

**Affiliations:** 1 School of Soil and Water Conservation, Beijing Forestry University, Beijing 100083, China; 2 Wuhan Linshui Engineering Consulting Co., Ltd, Wuhan 430070, China; 3 Beijing Zhongjiao Lvtong Technology Co., Ltd., Beijing 100010, China; 4 Plant Development Center for Soil and Water Conservation, Beijing 100038, China

**Keywords:** CASA model, Net ecosystem productivity, Remote sensing, Field data

## Abstract

Sea buckthorn (*Hippophae rhamnoides* L.)-based ecological engineering is critical for ecological restoration and carbon sequestration enhancement in China's Pisha sandstone region. However, accurate large-scale carbon sequestration quantification remains challenging: complex terrain, limited accessibility, and high spatial heterogeneity impede extensive field surveys. To address this, we developed an integrated framework combining multi-temporal remote sensing inversion and systematic field measurements. Using the Carnegie-Ames-Stanford Approach (CASA) model, we estimated 2013–2023 vegetation net primary productivity (NPP) and derived net ecosystem productivity (NEP) as a proxy for carbon sequestration density. Remote sensing-derived estimates were validated against *in situ* measurements across plots of varying stand ages (5–10 years) and site types (shady/sunny slopes, gully bottoms). Results showed strong agreement between remote sensing and field-measured carbon storage (*R^2^* = 0.79, RMSE = 25.07 tCO_2_ ha^−1^), confirming the model's reliability for regional carbon sink quantification in these shrublands. Spatially, sea buckthorn stands had higher carbon sequestration density in the study area's eastern versus western portion, with gully-bottom sites showing significantly higher capacity than slope sites (*p* < 0.05). This study validates the CASA model's effectiveness for sea buckthorn carbon sink monitoring in the fragile Pisha sandstone region, providing a species-specific remote sensing-field integrated pathway for carbon quantification in arid/semi-arid sea buckthorn ecosystems to support China's 'Dual Carbon' strategic goals.

## Introduction

Global climate change has driven a marked increase in the frequency of extreme weather events and progressive degradation of global ecosystems, presenting fundamental challenges to sustainable development worldwide. In response to these pressing challenges, reducing atmospheric CO_2_ concentrations and enhancing the carbon sequestration capacity of terrestrial ecosystems have emerged as widely adopted core strategies in global climate change mitigation efforts^[1−4]^. Aligned with the Paris Agreement's global climate action framework^[5]^, China formally announced its commitment to the 'Dual Carbon' strategic goals in September 2020, which target achieving carbon peak by 2030 and carbon neutrality by 2060^[6]^. A core measure for delivering these targets is carbon sink enhancement through ecological restoration. Since the late 20^th^ century, China has launched and implemented a series of large-scale ecological restoration projects—including the Grain-for-Green Program and the Natural Forest Protection Program—that have markedly increased regional vegetation cover, mitigated soil erosion, and enhanced ecosystem carbon sink capacity^[7,8]^. These initiatives constitute critical pathways for aligning ecological security with national climate action goals. Accurate quantification of the carbon sequestration contributions of key restoration species in ecologically fragile regions is therefore essential for rigorously quantifying and optimizing the effectiveness of these pathways.

The Pisha sandstone region is a well-recognized, highly representative ecologically fragile zone both within China and globally. Characterized by loosely consolidated strata and extremely low erosion resistance, this region is subject to severe soil erosion and persistent ecological degradation^[9]^. Located in the upper and middle reaches of the Yellow River, this region's ecological condition directly governs the water-sediment dynamics and ecological security of the river's middle and lower basins^[10]^. To mitigate land degradation and restore degraded ecological functions, sea buckthorn has been extensively planted and promoted as a pioneer species since the 1990s, widely recognized for its drought tolerance, adaptability to nutrient-poor barren soils, a robust and well-developed root system, and nitrogen-fixing capacity^[11,12]^. Its deep root architecture and nitrogen-fixing capacity promote the accumulation of soil organic carbon^[13]^, rendering it a high-potential carbon sink species in addition to its core erosion control function. Accordingly, this species has become one of the foundational species for ecological restoration in the Pisha sandstone region. While sea buckthorn-based ecological restoration projects have been widely demonstrated to be highly effective in erosion control, soil quality improvement, and vegetation recovery^[14]^, the carbon sequestration potential of these sea buckthorn stands as a substantial carbon sink remains to be systematically and comprehensively quantified. Against the backdrop of China's 'Dual Carbon' strategic goals, an urgent need exists to accurately quantify the carbon sequestration capacity of sea buckthorn stands and elucidate their carbon sink dynamics—critical foundational steps for optimizing the design of ecological restoration engineering and maximizing the ecosystem service benefits of these initiatives.

Traditional field-based surveys have long served as the primary method for ecosystem carbon sink quantification, delivering high measurement precision at the plot scale, but with critical limitations when applied to spatially complex, highly heterogeneous regions. The rugged terrain and highly variable site conditions in the Pisha sandstone region severely limit the extrapolation of data from limited sampling plots to broader regional scales^[15,16]^. Furthermore, while the carbon sequestration capacity of shrub ecosystems changes progressively with stand age, most existing studies fail to resolve the interannual dynamics and long-term trends of carbon density in these systems^[17,18]^. These challenges are compounded by the fact that field surveys are time-consuming, labor-intensive^[19−21]^, and poorly suited for large, inaccessible areas or steep terrain. In contrast, the Carnegie-Ames-Stanford Approach (CASA) model—driven by coupled remote sensing and meteorological data—enables continuous, large-scale, long-term estimation of net primary productivity (NPP) and net ecosystem productivity (NEP), directly addressing the spatiotemporal limitations of field surveys in complex topographic settings.

Recent advances in remote sensing technology have opened new avenues for large-scale carbon sink quantification. In particular, light use efficiency (LUE) models driven by multi-source satellite data have been widely applied to estimate net primary productivity (NPP) and net ecosystem productivity (NEP), providing a robust framework for characterizing the spatiotemporal patterns of ecosystem carbon uptake^[22−24]^. As a core metric of regional net carbon sequestration^[25,26]^ NEP is theoretically well-suited for quantifying sea buckthorn carbon sequestration capacity. However, the application of this approach in the Pisha sandstone region remains in its early stages, with no systematic validation against multi-dimensional *in situ* field measurements. Its estimation accuracy and reliability therefore require further optimization, particularly given the strong seasonal dynamics of carbon fluxes in temperate terrestrial ecosystems^[27]^.

These challenges are further compounded by the unique ecophysiological traits of sea buckthorn stands—including rapid growth, deep root architecture, and slow litter decomposition rates—that distinguish their carbon accumulation mechanisms from those of natural forests or grasslands. Moreover, the unique geological and climatic setting of the Pisha sandstone region likely modulates carbon fixation processes via its regulation of soil moisture, nutrient availability, and soil microbial activity. Accordingly, the development of a tailored remote sensing estimation framework for sea buckthorn carbon sinks is critical to address this key research gap, while also providing methodological insights for carbon accounting in other ecologically fragile ecosystems.

While carbon sink enhancement is not the primary objective of sea buckthorn-based ecological restoration projects, the large-scale afforestation delivered through these initiatives generates substantial carbon sequestration co-benefits. In this study, we use the sea buckthorn restoration initiative in the Pisha sandstone region as a representative case study for integrated ecosystem restoration aligned with China's dual-carbon strategic goals. We integrated multi-temporal Landsat imagery, long-term *in situ* meteorological data, digital elevation model (DEM) products, and *in situ* field sampling measurements to drive the Carnegie–Ames–Stanford Approach (CASA) model for net primary productivity (NPP) estimation. These NPP outputs were further coupled with a soil heterotrophic respiration model to simulate net ecosystem productivity (NEP), enabling robust quantification of regional carbon sequestration density. We established systematic validation plots spanning varying stand ages (5–10 years) and typical site types (shady slopes, sunny slopes, and gully bottoms), and measured carbon sequestration density across all ecosystem compartments—including aboveground biomass, belowground biomass, litter, and mineral soil layers—to construct a ground-truth dataset for validating our remote sensing inversion results.

This study aims to: (1) calibrate and validate the CASA model for sea buckthorn plantations using field data; (2) quantify spatiotemporal patterns of NEP and carbon sequestration across stand ages and site types; (3) assess uncertainties and carbon leakage in the estimation framework. The results are expected to provide a methodological reference for assessing carbon sink functions in sea buckthorn ecosystems across arid and semi-arid regions, and to support science-based management of ecological restoration projects in the context of China's dual-carbon goals.

## Materials and methods

### Study area

**Figure 1:**
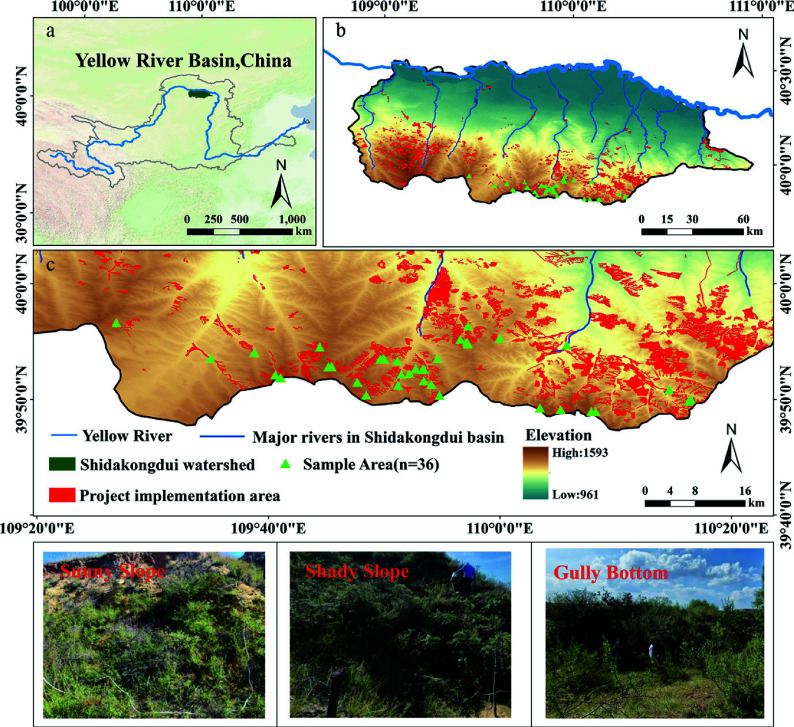
Study area and sample plot distribution. (a) Geographical location of the Shidakongdui Basin in the Yellow River Basin; (b) Topography, river system and distribution of ecological restoration projects; (c) Detailed distribution of sampling sites and project implementation areas. Data sources: the digital elevation model (DEM) was derived from the ALOS World 3D (AW3D) 12.5 m dataset (Japan Aerospace Exploration Agency, JAXA, https://nasadaacs.eos.nasa.gov/); the administrative boundary and river network data were obtained from the Resource and Environment Science and Data Center (RESDC), Chinese Academy of Sciences (https://resdc.cn/).

As shown in Fig. 1, the Dakuodui River originates in the Ordos Plateau, geographically located between 39°38′–40°33′ N and 108°50′–110°57′ E. Its upper reaches cross a loess hill-gully area dominated by Pisha sandstone landforms, the middle section passes through an aeolian sand zone, and the lower reaches form an alluvial floodplain before eventually converging into the Yellow River. The elevation of the watershed ranges from approximately 900 to 1,600 m, with overall terrain descending from west to east and from south to north. Over the long term, the exposed Pisha sandstone formations—typically appearing as interbedded red and white layers—have remained sparsely vegetated. These materials become sandy when dry and muddy when wet, contributing to severe surface instability and erosion. In recent years, China has launched sea buckthorn afforestation projects to control soil erosion in the Pisha sandstone area. Among them is the 'Ten Major Kongdui Sea Buckthorn Ecological Sediment Reduction Project', implemented from 2013 to 2019 in the Jin-Shan-Meng Pisha sandstone region, which serves as a key case study in this research.

### Data sources and preprocessing

All data processing in this study was conducted on the Google Earth Engine (GEE) platform. Datasets were accessed and processed through direct code calls, with key preprocessing steps—including atmospheric correction, cloud masking, and image compositing—performed directly within GEE. The primary remote sensing data source was Landsat 8 surface reflectance imagery (2013–2023), used for calculating annual vegetation indices (e.g., NDVI) and fraction of absorbed photosynthetically active radiation (FPAR). To ensure temporal continuity and to fill data gaps in earlier years, Landsat 7 ETM+ imagery (2008–2013) was also incorporated. The annual maximum NDVI series from 2013 to 2023 was derived from the harmonized Landsat 5/7/8 archive after smoothing and aggregation. Additionally, meteorological datasets—including near-surface air temperature, cumulative precipitation, mean annual temperature, solar radiation, and evapotranspiration—were spatially interpolated and resampled to align with the 30 m resolution of the Landsat imagery. Detailed data specifications, including temporal coverage and resolution, are provided in Table 1.

**Table 1:** Data sources.

Data classes	Products	Temporal resolution	Spatial resolution
Landsat 8 OLI	LANDSAT/LC08/C02/T1_L2	16 d	30 m
Landsat 7 ETM+	LANDSAT/LE07/C02/T1_L2	2008–2013	30 m
Meteorological data	ECMWF ERAS-Land	30 d	11,132 m
DEM	ALOS	Static	12.5 m
NDVI	LANDSAT/LC08/C02/T1_L2	16 d	30 m

A Digital Elevation Model (DEM) with a spatial resolution of 12.5 m, acquired by the ALOS satellite, was obtained from the NASA Earth Science Data website (https://nasadaacs.eos.nasa.gov) and used to represent the topography of the study area. Slope and aspect were extracted from the DEM in ArcMap, which subsequently supported the stratification of carbon layers and site-type classification for the planted sea buckthorn communities.

### Research methods

#### Estimation of carbon sequestration density by remote sensing

The Carnegie-Ames-Stanford Approach (CASA) model is one of the most widely used methods for estimating regional net primary productivity (NPP). Operating on the principle of light-use efficiency, the model calculates NPP based on solar radiation and the photosynthetically active radiation (PAR) absorbed by vegetation^[28]^. The main calculation formula of the model is as follows:

1 $ {NPP}\left({x,t}\right)={APAR}\left({x,t}\right)\times \varepsilon \left({x,t}\right) $

In the equation: *NPP*(*x*,*t*) represents the NPP value at pixel *x* in month t(gC·m^−2^); *APAR*(*x*,*t*) denotes the photosynthetically active radiation absorbed at pixel *x* in month *t* (MJ·m^−2^), and *ε*(*x*,*t*) is the actual light use efficiency at pixel *x* in month *t* (gC·MJ^−1^).

2 ${APAR}\left({x,t}\right)={SOL}\left({x,t}\right)\times {FPAR}\left({x,t}\right)\times {0.48} $

where, *SOL*(*x*,*t*) represents the total solar radiation at pixel x in month t (MJ·m^−2^); *FPAR*(*x*,*t*) denotes the fraction of photosynthetically active radiation absorbed by the vegetation layer. The constant 0.48 represents the proportion of total solar radiation that can be utilized as effective radiation by sea buckthorn vegetation.

3 $ {FPAR}\left({x,t}\right)=\dfrac{\left({NDVI}\left({x,t}\right){-NDV}{{I}}_{{i,\min}}\right)}{\left({NDV}{{I}}_{{i,\max}}{-NDV}{{I}}_{{i,\min}}\right)}\times \left({FPA}{{R}}_{\max }{-FPA}{{R}}_{\min }\right)+{FPA}{{R}}_{\min } $

where, *NDVI*(*x*,*t*) represents the vegetation index at pixel *x* in month *t*; *NDVI*_*i*,max_ and *NDVI*_*i*,min_ denote the theoretical maximum and minimum NDVI values for shrubland (specifically sea buckthorn), respectively, and are not derived from the actual NDVI range of the study area. In this study, the theoretical values for shrub vegetation provided by Zhu et al.^[29]^ were adopted, with *NDVI*_*i*,max_ = 0.636, and *NDVI*_*i*,max_ = 0.023. Similarly, *FPAR*_max _and *FPAR*_min_ are vegetation-independent constants, set to 0.95 and 0.001, respectively.

4 $ \mathrm{NDVI}=\dfrac{{S}{{R}}_{{B5}}{- S}{{R}}_{{B4}}}{{S}{{R}}_{{B5}}{+ S}{{R}}_{{B4}}} $

The actual light use efficiency (*ε*) is defined as the maximum light use efficiency under ideal conditions without environmental stress, adjusted for constraints imposed by temperature and moisture. The model is expressed as follows:

5 $\varepsilon \left({x,t}\right)={{T}}_{{\varepsilon 1}}\left({x,t}\right)\times {{T}}_{{\varepsilon 2}}\left({x,t}\right)\times {{W}}_{\varepsilon}\left({x,t}\right)\times {\varepsilon}_{\max }$

where, ${{T}}_{{\varepsilon 1}}\left({x,t}\right) $ represents the low-temperature stress factor on light use efficiency; ${{T}}_{{\varepsilon 2}}\left({x,t}\right) $ denotes the high-temperature stress factor; *W*_*ε*_(*x*,*t*) refers to the water stress factor; *ε*_max_ is the maximum light use efficiency under ideal non-stressed conditions. In this study, the value for shrublands provided by Zhu et al.^[29]^ was adopted, with *ε*_max_ = 0.429 gC·MJ^−1^.

6 $ {{T}}_{{\varepsilon 1}}({x},{t})={0.8}+{0.02}\times {{T}}_{\mathrm{opt}}({x})-{0.0005}\times [{{T}}_{\mathrm{opt}}\left({x}\right){{]}}^{{2}} $

where, ${{T}}_{\mathrm{opt}}({x}) $ represents the optimum temperature for vegetation growth, generally defined as the temperature at which NDVI reaches its maximum. In this study, a uniform value of 22 °C was adopted for this parameter across all pixels and time periods.

7 \begin{equation*}\begin{split}{{T}}_{{\varepsilon 2}\left({x,t}\right)}=& 1.184/\left\{1 +\exp \left[0.2 \times\left({{T}}_{\mathrm{opt}}\left({x}\right){- 10-T}\left({x,t}\right)\right)\right]\right\}\\
&\times \dfrac{{1}}{\left\{{1 +}\exp \left[0.3 \times\left(-{{T}}_{\mathrm{opt}}\left({x}\right){- 10 +T}\left({x,t}\right)\right)\right]\right\}} \end{split}\end{equation*}

where, *T*(*x*,*t*) represents the temperature value at pixel *x* in month *t* (°C).

8 $ {{W}}_{\varepsilon}\left( {x,t}\right) ={0.5}+{0.5}\times \dfrac{{E}\left( {x,t}\right) }{{{E}}_{{p}}\left( {x,t}\right) } $

where, *E*(*x*,*t*) represents the actual evapotranspiration in the region (mm); *E*_*p*_(*x*,*t*) denotes the potential evapotranspiration in the region (mm).

9 $ {E}\left({x},{t}\right)=\dfrac{P\cdot{{R}}_{{n}}\times({{P}}^{{2}}{+R}_{{n}}^{{2}}+P\times{{R}}_{{n}}{)}}{{(P+}{{R}}_{{n}})\times({{P}}^{{2}}+R_{n}^{2})} $

where, *P* denotes the precipitation amount (mm); *R_n_* represents the net radiation (MJ·m^−^^2^).

10 $ {{E}}_{{p}}\left({x,t}\right) ={0.0023}\times \left({T}\left({x,t}\right) {+ 17.8}\right) \times {{R}}_{{n}}\times {30} $

Net ecosystem productivity (NEP) represents the residual organic matter after subtracting soil heterotrophic respiration from net primary productivity (NPP), serving as a direct measure of regional carbon sequestration density. The calculation formula is as follows:

11 $ {NEP}={NPP}-{{R}}_{{h}} $

where, *R_h_* represents the consumption rate of soil organic matter by heterotrophic respiration (gC·m^−^^2^), calculated using the following formula:

12 $ {\ln}{{R}}_{{h}}={0.22}+{0.87}\times {\ln}{{R}}_{{s}} $

where, *R_s_* denotes the monthly soil respiration rate (gC·m^−^^2^), derived from the following equation:

13 ${{R}}_{{s}}={f}\times {{e}}^{(b\times{{{T}}_{{a}}}{)}}\times [{P}/({k}+{P})] $

where, *b* is the temperature sensitivity coefficient of soil respiration, with a value of 0.05452; *T_a_* represents the monthly average temperature (°C); *P* indicates the monthly precipitation (cm); *f* and *k* are constants, with values of *f* = 1.250, and *k* = 4.259^[6]^. In this study, the multi-year cumulative NEP was adopted to represent the net carbon sequestration density over the corresponding period.

#### Plot survey and carbon sequestration density calculation

Based on DEM, NDVI, and remote sensing imagery of the project area, sea buckthorn forests were stratified by community age, site type, and vegetation density. Site types were classified into three categories: shaded slopes (slope 5°–25°, aspect 0°–90° and 270°–360°), sunny slopes (slope 5°–25°, aspect 90°–270°), and gully bottoms (slope < 5°). Stand age vectors were delineated using historical planting records of sea buckthorn, while vegetation cover was divided into three levels based on NDVI using the natural breaks method.

A total of 36 sample plots representing all combinations of these categories were randomly selected for field investigation (Table 2). Each plot contained two replicated 5 m × 5 m sea buckthorn quadrats (experimental group), intentionally distributed across varying slope gradients to capture topographic heterogeneity and reduce systematic bias. Within each experimental quadrat, two 1 m × 1 m subplots were established for monitoring herbaceous plants and litter. Additionally, three soil sampling points were randomly arranged per plot. In total, the experimental setup included 72 sea buckthorn quadrats, 144 herbaceous and litter subplots, and 108 soil sampling points.

**Table 2:** Sample information for *Hippophae rhamnoides* L. plantation stand community surveys.

Community age (years)	Herb cover (%)	Stand density (plant·ha^−1^)	Sample plots	Experimental plots	Control plots	Herbaceous and litter subplots	Soil sampling points
10	52−88	2,400−11,600	7	14	7	42	28
9	65−75	7,200−10,800	4	8	4	24	16
8	55−85	2,800−11,600	6	12	6	36	24
7	36−86	5,200−15,600	7	14	7	42	28
6	60−92	7,200−10,800	6	12	6	36	24
5	58−69	7,600−10,000	6	12	6	36	24

To establish a baseline, native grassland vegetation was sampled under comparable site conditions as a reference^[30]^. The control group consisted of 72 herbaceous and litter subplots, and 36 soil sampling points.

Carbon content varies across different components of the sea buckthorn ecosystem—including above-ground biomass, below-ground biomass, herbaceous layer, litter layer, and soil—and was therefore assessed using a stratified biomass approach. This study applied the carbon content parameters for sea buckthorn organs established by Dang et al.^[31]^: leaf carbon content was 54.72%, branch and stem carbon content was 54.58%, and root carbon content was 45.26%. Given the small variation in carbon content between leaves and branches, the mean value of 54.65% was used for total above-ground biomass. Default carbon content values of 0.40 and 0.37 were adopted for the herbaceous and litter layers, respectively, in accordance with Clean Development Mechanism (CDM) guidelines. Net carbon sequestration density was calculated as the difference in actual carbon density between sea buckthorn plots and native dry grassland vegetation, with CO_2_ emissions from fuel-based machinery during afforestation activities deducted.

Above- and below-ground biomass measurement: within each sea buckthorn sample quadrat, the ground diameter, crown width, and height of each individual or clump were measured. Based on the average values, representative standard plants were selected and harvested for destructive sampling. Fresh weights of stems, branches, leaves, and roots were recorded. Due to the rhizomatous growth habit of sea buckthorn, which prevents accurate separation of individual root systems, all roots within a 30 cm radius from the center of each standard plant were excavated. Biomass models for above- and below-ground components were developed by correlating measured biomass with morphometric parameters (ground diameter, crown width, and height), enabling non-destructive biomass estimation for other individuals.

Herbaceous and litter biomass measurement: All herbaceous plants (including roots) and litter within each 1 m × 1 m subplot were collected and weighed to determine total fresh weight. A representative fresh sample of 100–500 g was taken to the laboratory, oven-dried at 80 °C to a constant weight, and used to calculate dry weight and dry-to-fresh ratio. Total herbaceous biomass per unit area was then estimated based on subplot area. Similarly, litter dry biomass per unit area was derived by applying the dry-to-fresh ratio to the total litter fresh weight.

Soil carbon content measurement: soil samples were collected from 0–10, 10–20, and 20–50 cm depths at each sampling point using cutting rings and aluminum boxes. After removing roots and plant debris, samples were air-dried for soil organic carbon analysis, and bulk density was determined from the ring samples (Table 3). Soil moisture content was measured by the direct drying method, and soil organic carbon density for each layer was calculated as:

14 $ {SOC}{{D}}_{{i}}={SO}{{C}}_{{i}}\times {{p}}_{{i}}\times {H}\times {10} $

where, *i* represents the soil layer depth intervals: 0–10, 10–20, and 20–50 cm; *SOCD_i_* denotes the soil organic carbon density of the *i*-th layer (gC·m^−2^); *SOC_i_* is the soil organic carbon content of the *i*-th layer (g·kg^−1^); *ρ_i_* refers to the soil bulk density of the *i*-th layer (g·cm^−3^); *H* is the thickness of the soil layer (cm). The coefficient 10 is used for unit conversion.

**Table 3:** Soil characteristics of *Hippophae rhamnoides* L. plantation stands across different stand ages.

Soil depth	Soil index	Unit	Community age					
			10	9	8	7	6	5
0−10 cm	BD	g/cm^3^	1.25 ± 0.17a	1.24 ± 0.24a	1.27 ± 0.14a	1.21 ± 0.35a	1.26 ± 0.21a	1.18 ± 0.21a
	SOC	g/kg	6.2 ± 2.79a	4.44 ± 2.79b	3.48 ± 1.52b	3.12 ± 1.39b	3.76 ± 2.13b	3.04 ± 1.62b
10−20 cm	BD	g/cm^3^	1.29 ± 0.18a	1.26 ± 0.29a	1.42 ± 0.59a	1.26 ± 0.24a	1.24 ± 0.15a	1.26 ± 0.15a
	SOC	g/kg	4.49 ± 2.07a	3.37 ± 1.96ab	2.56 ± 1.18bc	1.92 ± 0.31c	2.22 ± 1.11bc	2.3 ± 0.91bc
20−50 cm	BD	g/cm^3^	1.25 ± 0.11a	1.16 ± 0.27a	1.31 ± 0.16a	1.16 ± 0.34a	1.28 ± 0.15a	1.24 ± 0.14a
	SOC	g/kg	4.46 ± 2.53a	4.52 ± 3.36a	2.69 ± 2.22b	2.08 ± 0.98b	2.06 ± 0.75b	2.06 ± 0.75b

## Results

### NEP estimation results of sea buckthorn plantation

**Figure 2:**
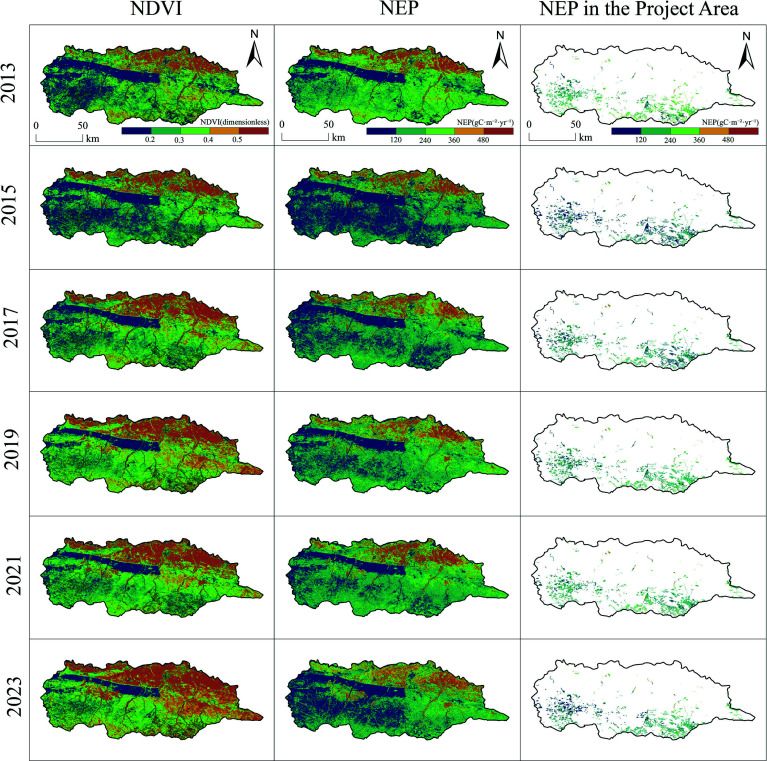
Spatial distribution patterns of NDVI, NPP, and NEP in the study area, 2013–2023.

Figure 2 illustrates the spatial distribution patterns of NDVI and NEP across the study area, as well as NEP within the project area, from 2013 to 2023. In terms of NDVI distribution, significant spatial heterogeneity is observed. High NDVI values were primarily concentrated in the central and southern regions, while the northern areas consistently exhibited lower values. Between 2013 and 2023, the extent of high-NDVI areas in the central and southern zones gradually expanded, with a notable increase in the spatial continuity of high vegetation coverage after 2015, reflecting sustained improvement in vegetation growth conditions.

This improvement in vegetation cover and vigor directly contributed to enhanced carbon sequestration. The spatial distribution of NEP showed a strong correspondence with that of NDVI, with high NEP values likewise concentrated in the well-vegetated central and southern regions. Temporally, the spatial pattern of NEP evolved in tandem with vegetation dynamics: high-NEP zones were fragmented during 2013–2015 but became significantly more continuous between 2019 and 2023. This spatiotemporal evolution underscores that the recovery and expansion of vegetation, as captured by increasing NDVI, were the primary drivers behind the strengthening and spatial consolidation of the carbon sink in these sea buckthorn plantations.

Furthermore, the spatial distribution of NEP within the project area reveals a gradual increase in the number and coverage of high-value patches from 2013 to 2023, underscoring the positive regulatory role of the ecological project in shaping the regional carbon sequestration pattern.

**Figure 3:**
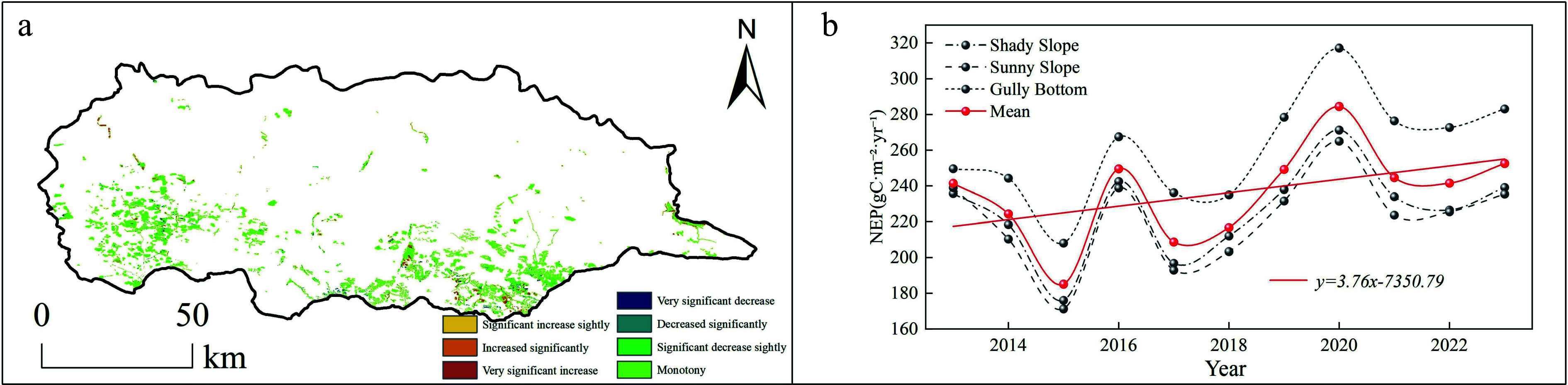
Spatio-temporal variation trends of NEP in the study area. (a) Spatial distribution of NEP change trends in *Hippophae rhamnoides* L. plantation stands 2013–2023; (b) temporal variation trend of NEP in *Hippophae rhamnoides* L. plantation stands 2013–2023.

Figure 3 presents the spatio-temporal evolution of net ecosystem productivity (NEP) from two perspectives: spatial change patterns, and temporal trends. In terms of spatial variation types, NEP remained largely stable across most of the study area, accounting for 81% of the total, indicating no significant fluctuation in carbon sink capacity in these regions. Areas showing an increasing trend in NEP constituted 5.3%, while those exhibiting a decreasing trend accounted for 7.5%. These patterns reflect a generally stable carbon sequestration background, accompanied by localized variations in carbon uptake dynamics.

The interannual variation in NEP from 2013 to 2023 was further analyzed across different site types, with mean values used to establish temporal trends. Overall, NEP showed considerable interannual fluctuation but a statistically significant positive trend, characterized by a linear coefficient of 2.63. Furthermore, gully-bottom sites demonstrated significantly higher NEP than both shady and sunny slopes (*p* < 0.05), whereas no significant difference was observed between shady and sunny slopes (*p* > 0.05).

### Construction of sea buckthorn growth model

#### Growth under different stand ages and site types

**Figure 4:**
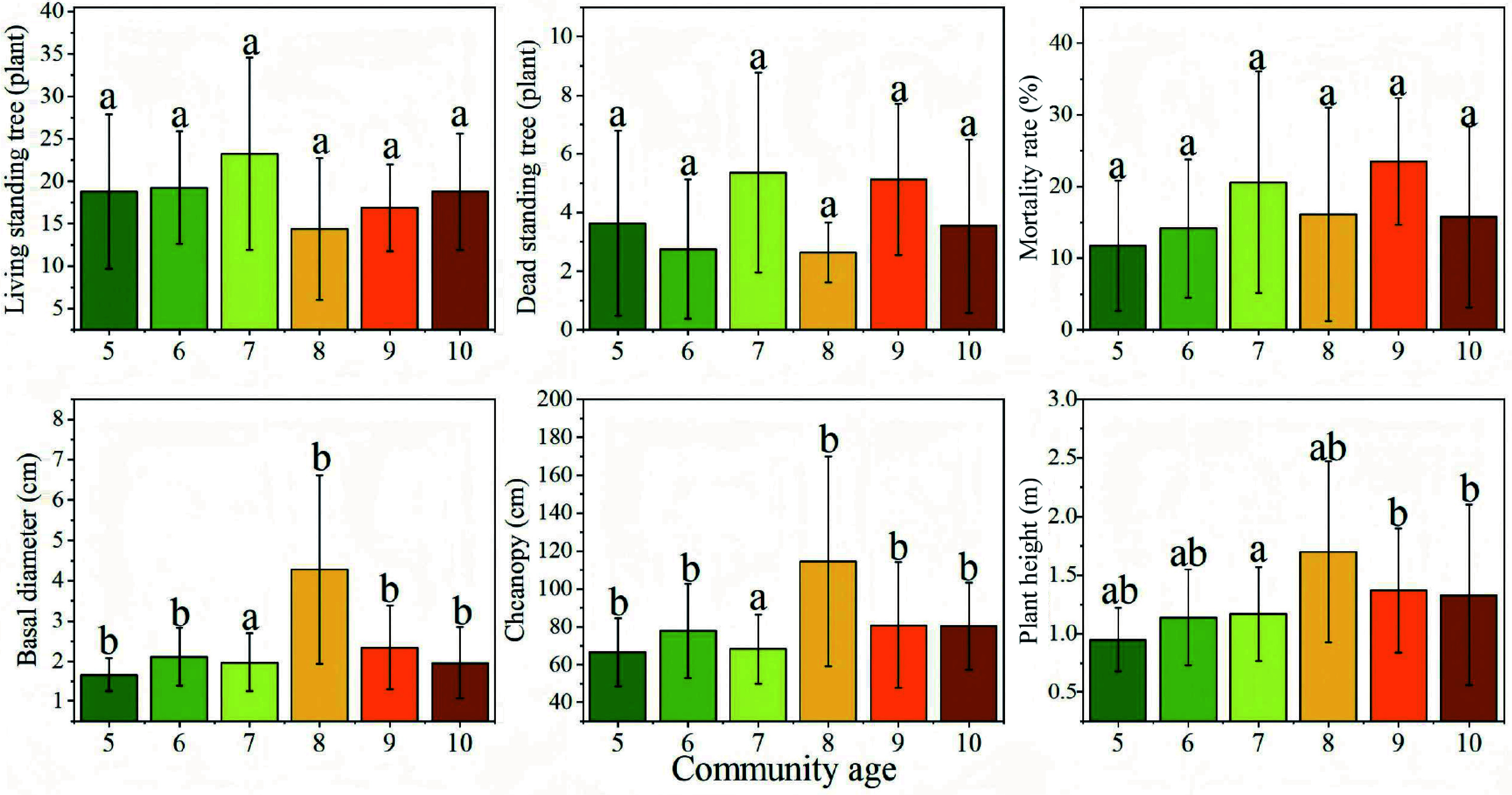
Growth characteristics of *Hippophae rhamnoides* L. plantation stands across different stand ages. *n* = 36 sample plots.

The statistical results of growth factors across different years are presented in Fig. 4. No significant differences or consistent patterns were observed in the numbers of live and dead standing trees in most years. However, both live and dead tree counts were notably higher in 2016, possibly due to environmental stresses specific to that year. Base diameter reached its maximum in 2015, significantly exceeding that in other years (*p* < 0.05). Crown width and plant height generally increased with stand age, peaking in the 8-year-old stands (2015), suggesting that this stage may represent the optimal growth phase for sea buckthorn.

**Figure 5:**
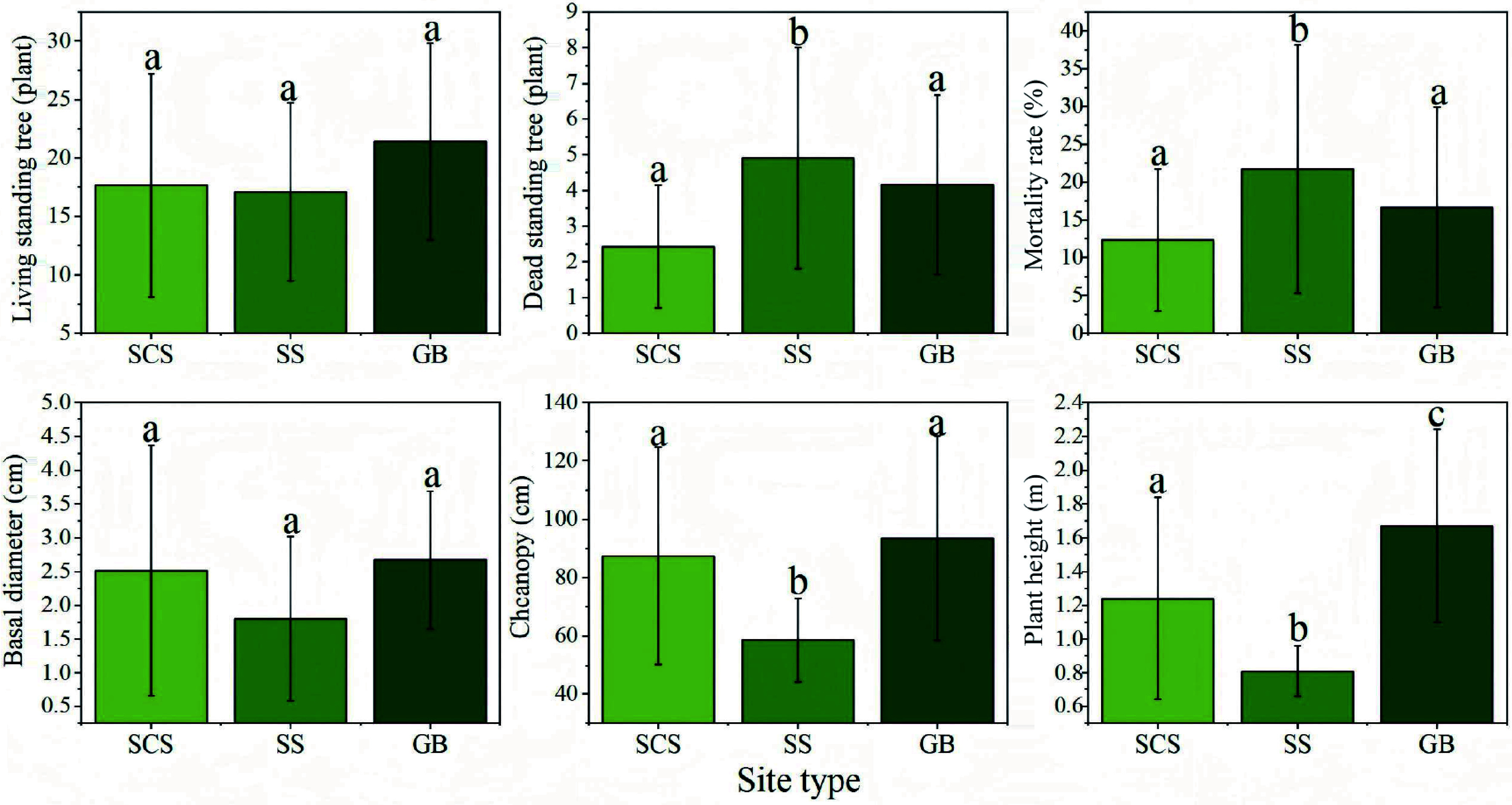
Growth characteristics of *Hippophae rhamnoides* L. plantation stands across different site types. *n* = 36 sample plots. SCS: shady slope; SS: sunny slope; GB: gully bottom.

Growth characteristics under different site types are also summarized in the corresponding Fig. 5. Site conditions significantly influenced sea buckthorn growth and survival. Shady slopes and gully bottoms supported better growth, reflected in higher numbers of live trees and lower mortality rates. In contrast, sunny slopes exhibited significantly higher mortality, indicating stronger environmental stress. Morphologically, gully-bottom shrubs achieved the largest base diameter, crown width, and plant height, differing significantly from those on sunny slopes (*p* < 0.05)—likely a result of more favorable moisture conditions and moderate light. High light intensity on sunny slopes may exacerbate soil water evaporation, suppressing plant growth and elevating mortality risk. Additionally, crown width and plant height on shady slopes were significantly greater than those on sunny slopes (*p* < 0.05).These findings highlight the importance of site selection in afforestation planning, suggesting that prioritizing gully bottoms and shaded slopes can enhance plantation survival, growth, and overall carbon sequestration potential.

#### Biomass model construction

**Figure 6:**
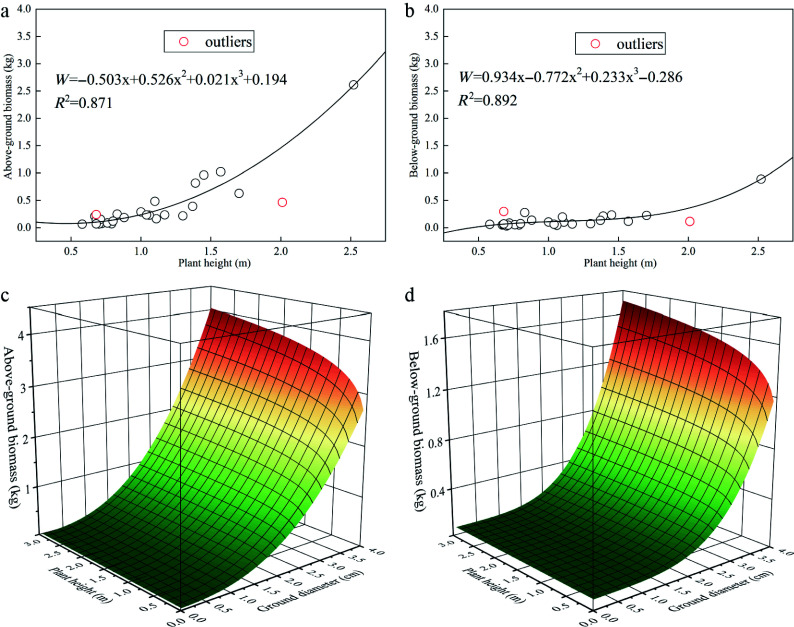
Optimal single- and dual-factor growth models for *Hippophae rhamnoides* L. *n* = 36 sample plots. (a) Single-factor growth model of above-ground biomass; (b) Single-factor growth model of below-ground biomass; (c) Dual-factor growth model of above-ground biomass; (d) Dual-factor growth model of below-ground biomass.

Based on the growth characteristics of sea buckthorn across different stand ages and site types, a total of 28 standard plants were harvested. After excluding two outliers that deviated significantly from the cluster, we developed both single- and dual-factor growth models for above- and below-ground biomass using various fitting equations. By comparing the goodness of fit (*R^2^*) and considering operational feasibility among single-, double-, and triple-factor models, the optimal single- and dual-factor growth models for sea buckthorn were identified, as summarized in the Table 4 and Fig. 6. To more accurately model the above- and below-ground carbon sequestration density in planted sea buckthorn forests across different stand ages, the dual-factor growth model—which demonstrated superior fit—was selected for further analysis. (Additional fitted models are provided in the Supplementary Tables S1−S4).

**Table 4:** Optimal single- and dual-factor growth models for *Hippophae rhamnoides* L.

		Model	*R^2^*
Single-factor	Above-ground biomass	*W*_*t*_ = −0.503*H* + 0.526*H*^*2*^ + 0.021*H*^*3*^ + 0.194	0.871
	Below-ground biomass	*W*_*d*_ = 0.934*H* − 0.772*H*^*2*^ + 0.233*H*^*3*^ − 0.286	0.892
Dual-factor	Above-ground biomass	*W*_*t*_ = 0.228*D^0.1* × *H^2.488* + 0.006	0.923
	Below-ground biomass	*W*_*d*_ = 0.018*D^0.1* × *H^3.981* + 0.073	0.898
Where W_t_ and W_d_ represent above-ground and below-ground biomass (kg), respectively; D is the ground diameter (cm); and H denotes plant height (m).			

### Measured carbon sequestration density across stand ages and site types

**Figure 7:**
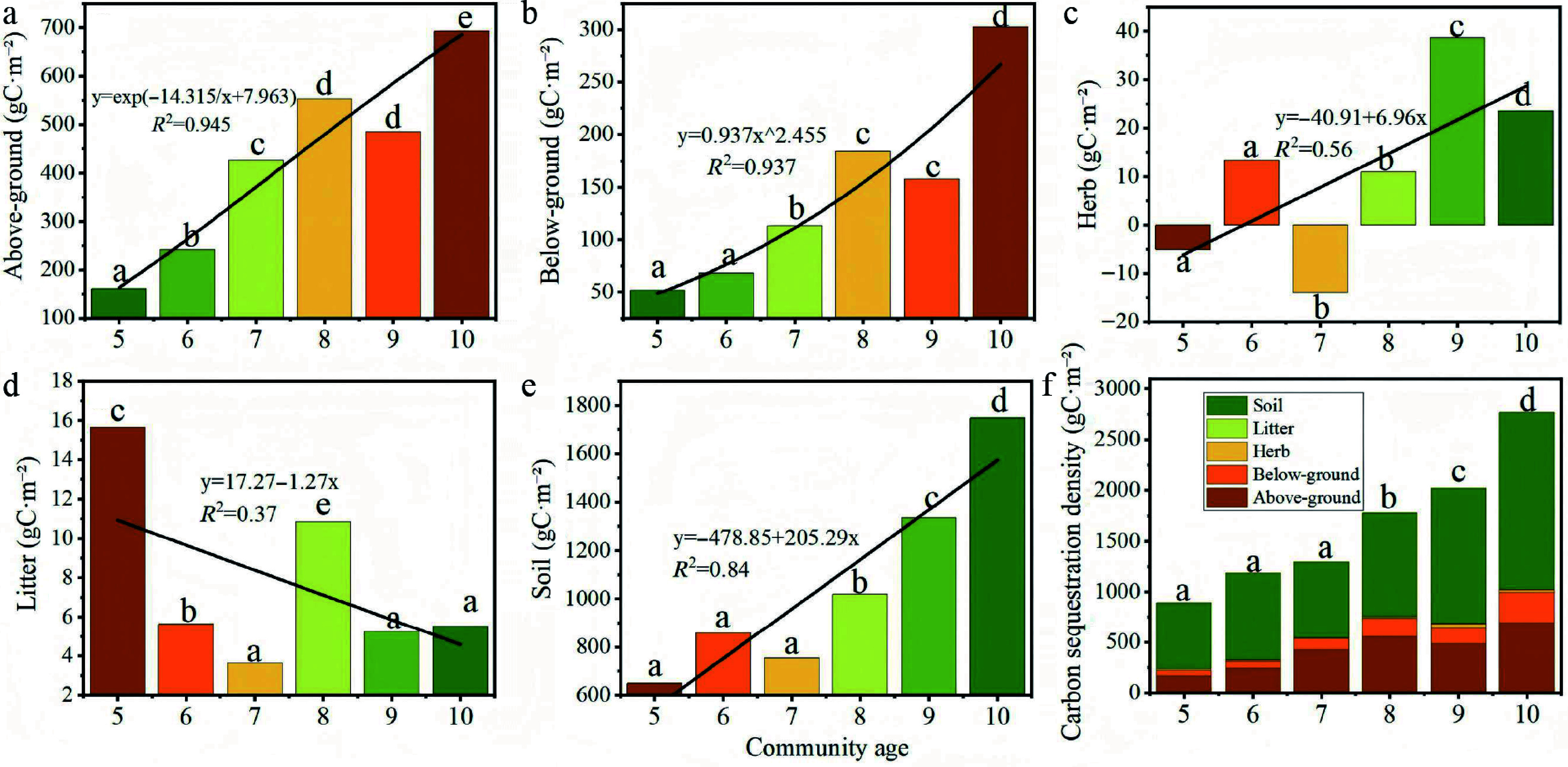
Distribution of carbon sequestration density in *Hippophae rhamnoides* L. plantation stands 2013–2018. (a) Above-ground carbon sequestration density; (b) below-ground carbon sequestration density; (c) herb layer carbon sequestration density; (d) litter layer carbon sequestration density; (e) soil carbon sequestration density, and (f) total ecosystem carbon sequestration density.

Figure 7 illustrates the carbon sequestration density across different stand ages. While no significant interannual differences were observed in above-ground biomass, its overall value increased with stand age. The 10-year-old stands exhibited the highest above-ground carbon density (692.55 gC·m^−2^), significantly exceeding the lowest value recorded in 5-year-old stands (168.91 gC·m^−2^). Similarly, below-ground biomass showed no significant annual variations but demonstrated a general increasing trend, also peaking in 10-year-old communities (302.80 gC·m^−2^).

The herbaceous layer carbon density followed a distinct pattern, initially decreasing from years 5 to 7 before increasing from years 7 to 10, with significant differences detected between age classes (*p* < 0.05). Notably, the value in 10-year-old stands (23.56 gC·m^−2^) was significantly higher than the negative value observed at year 7 (−13.99 gC·m^−2^), indicating a shift from carbon source to sink in the herbaceous layer over time. The litter layer reached its maximum carbon density at 5 years (15.63 gC·m^−2^), suggesting that litter gradually transitions to a carbon source as the sea buckthorn plantation matures. In contrast, the soil layer represented the largest carbon pool, with its sequestration density peaking at 10 years (1,746.36 gC·m^−2^).

Total ecosystem carbon storage increased significantly with stand age (*p* < 0.05). The 10-year-old stands contained the highest total carbon stock, showing significant differences from all younger age classes. The lowest carbon stock was found in 5-year-old stands, which were statistically similar to 6 and 7-year-old stands but significantly lower than 9 and 10-year-old stands.The magnitude of carbon accumulation, particularly in soil and mature stands, is comparable to, or exceeds that reported for other shrub ecosystems in arid and semi-arid regions, underscoring the effectiveness of sea buckthorn plantations as a carbon sink.

**Figure 8:**
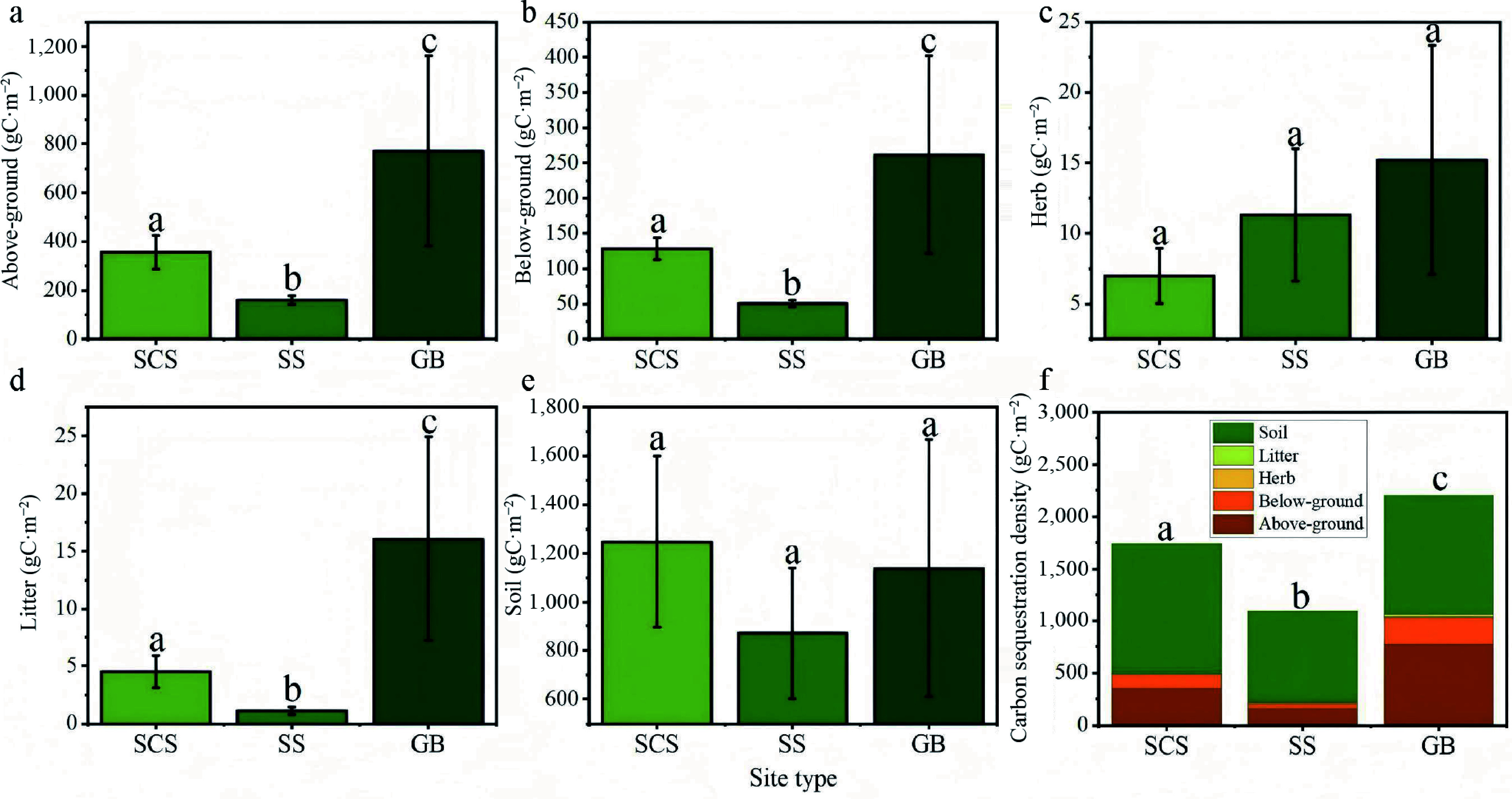
Carbon sequestration density of *Hippophae rhamnoides* L. plantation stands across different site types. (a) Above-ground carbon sequestration density; (b) below-ground carbon sequestration density; (c) herb layer carbon sequestration density; (d) litter layer carbon sequestration density; (e) soil carbon sequestration density, and (f) total ecosystem carbon sequestration density. SCS: shady slope; SS: sunny slope; GB: gully bottom.

Carbon sequestration density was categorized by site type and analyzed for differences, as illustrated in the accompanying Fig. 8. Overall, a consistent hierarchical pattern was observed: gully bottom > shaded slope > sunny slope. Both above- and below-ground biomass were significantly greater in gully bottoms than on sunny slopes (*p* < 0.05), a result attributable to more favorable moisture conditions in lower topographic positions.

The herbaceous layer also exhibited higher carbon storage in gully bottoms than on shaded and sunny slopes, though the differences were modest. This pattern likely reflects competitive interactions during early stand development, where vigorous sea buckthorn growth in gully settings suppressed herbaceous plant establishment. In contrast, the litter layer showed statistically significant variation across site types, whereas soil carbon storage did not differ significantly. With the exception of litter accumulation, which was notably lower on slopes, soil carbon density remained relatively uniform across site types, suggesting that site conditions exert limited influence on soil carbon dynamics at the scale of this study.

## Discussion

### Comparisons and uncertainties

In this study, we quantified the carbon sequestration density of sea buckthorn plantation stands in the Pisha sandstone region by simulating net ecosystem productivity (NEP) and calculating its multi-year cumulative carbon stock. Feng et al.^[32]^ implemented a modified CASA model to simulate net primary productivity (NPP) in the ecologically fragile Sichuan-Chongqing region, which features complex plateau-basin topography, and revealed the spatiotemporal patterns of vegetation carbon sequestration and its responses to climate change and anthropogenic activities. Gong et al.^[33]^ employed the CASA model to estimate actual net primary productivity (ANPP) across six typical ecologically fragile regions in China (e.g., the Loess Plateau and Southwest Karst region), and quantitatively disentangled the relative contributions of climate change and anthropogenic activities to vegetation degradation and restoration. Consistent with these prior studies, which have verified the reliability of the CASA model for carbon sink quantification in fragile ecosystems, our study further targets the unique lithological properties and arid climatic conditions of the Pisha sandstone region, with a focused analysis of the core restoration species sea buckthorn, to address a critical research gap in species-specific carbon sequestration quantification within these extremely fragile ecosystems.

**Figure 9:**
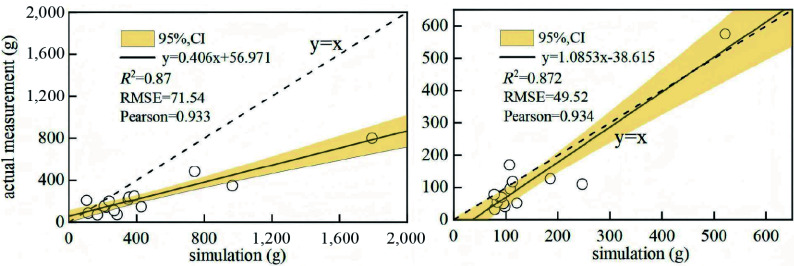
Goodness-of-fit validation for the *Hippophae rhamnoides* L. growth model.

The accuracy of the above-ground and below-ground biomass allometric models was validated using an independent dataset from 15 reference trees (aged 3–11 years) collected in the Huangshui River Basin, Qinghai Province, China^[34]^. As shown in Fig. 9, the validation sites in Qinghai Province, while geographically distinct from our study area, share the same Pisha sandstone lithology and are subject to comparable edaphic conditions and aridity stress. The high goodness-of-fit (*R^2^* = 0.87), therefore not only verifies the robustness of our developed biomass models, but also confirms their transferability to analogous ecologically fragile ecosystems. This transferability is likely driven by the consistent allometric growth patterns of sea buckthorn under semi-arid climatic conditions.

**Figure 10:**
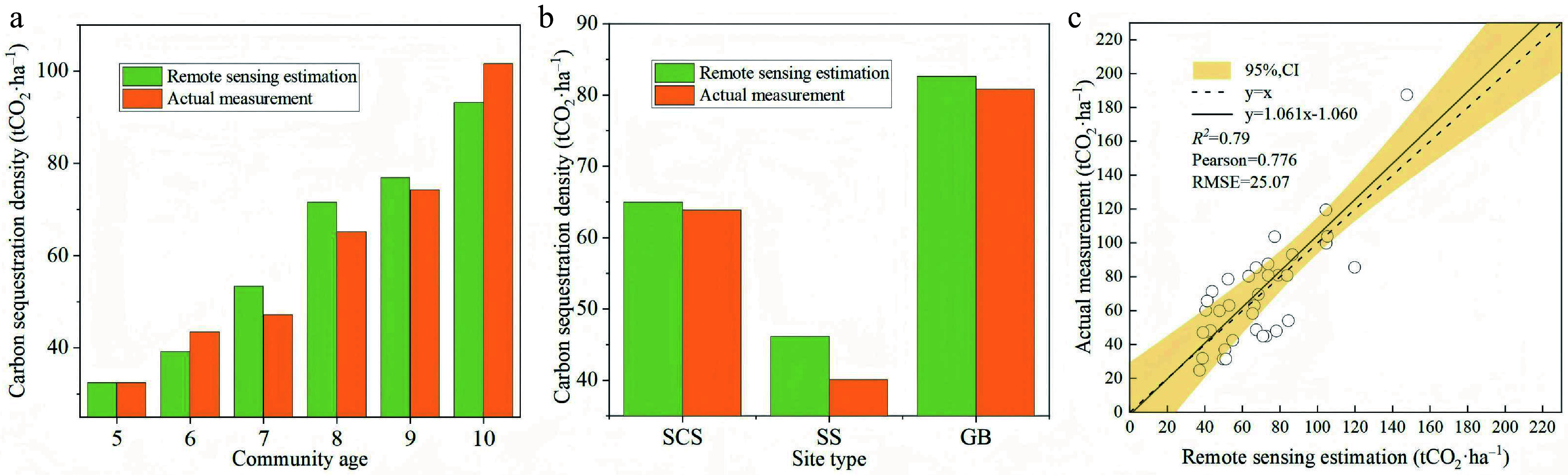
Goodness-of-Fit Between Remote Sensing-Derived and Field-Measured Carbon Sequestration Density in *Hippophae rhamnoides* L. Plantation Stands. (a) Comparison of remote sensing-derived and field-measured carbon sequestration density across different stand ages; (b) comparison of remote sensing-derived and field-measured carbon sequestration density across different site types; (c) comparison of remote sensing-derived and field-measured carbon sequestration density across all 36 sample plots. SCS: shady slope; SS: sunny slope; GB: gully bottom.

Furthermore, we validated the estimation accuracy of our remote sensing-derived NEP by comparing it with field-measured carbon sequestration densities from 36 sampling plots. As shown in Fig. 10, the two datasets exhibited strong agreement in both spatio-temporal distribution patterns and numerical magnitude. This consistency was particularly pronounced in sites with favorable moisture conditions (e.g., gully bottoms and shady slopes), where remote sensing-derived NEP closely matched field measurements. In contrast, remote sensing estimates were significantly higher than field-measured values at environmentally stressed sites such as sunny slopes. This overestimation is likely attributable to the model's water stress factor failing to fully capture the extreme soil drought conditions that characterize sunny slope sites, which leads to inflated estimates of actual light use efficiency (LUE) and subsequent overprediction of NEP in these areas.

The scatter plot of estimated vs measured carbon sequestration density (Fig. 10) shows that data points are distributed relatively evenly on both sides of the 1:1 line. Linear regression and Pearson correlation analysis revealed a strong positive correlation (*r* = 0.776), with a regression slope of 1.061, an *R^2^* of 0.79, and a root mean square error (RMSE) of 25.07 tCO_2_ ha^−1^. These results confirm a satisfactory model fit, demonstrating that the cumulative carbon sequestration density derived from NEP estimates has high quantitative accuracy.

The uncertainties in this study mainly originate from the following aspects:

Although Landsat-series satellite data provide a moderate spatial resolution suitable for quantifying vegetation cover, they cannot reliably differentiate *Hippophae rhamnoides* L. from co-occurring shrub species, particularly in early growth stages or areas with sparse vegetation cover. To mitigate this uncertainty, future work should integrate very-high-resolution satellite imagery with machine learning classifiers to improve species-level mapping accuracy, thereby enhancing the spatial precision of carbon sink estimates.

The maximum light use efficiency parameter (*ε*_max_) used in the CASA model was derived from a shrub-specific value reported by Zhu et al.^[29]^. While this value has been robustly validated for shrublands across China, it has not undergone *in situ* calibration for drought-adapted *H. rhamnoides* stands in the Pisha sandstone region^[35,36]^. Failure to implement species-specific, site-localized parameterization can introduce systematic biases in light use efficiency (LUE) simulations for semi-arid shrublands. Previous studies have demonstrated that coupling *in situ* photosynthetic measurements with remote sensing inversion is a robust approach for site-specific *ε*_max_ calibration in ecologically fragile ecosystems^[37]^. Additional uncertainty arises from the soil heterotrophic respiration model, which is highly sensitive to input temperature and precipitation data. The nonlinear response of soil respiration to soil moisture introduces further variability, particularly in the arid to semi-arid conditions of our study area^[38]^. Furthermore, the water stress factor in the CASA model cannot fully represent the extreme soil drought stress on sunny slopes, which is a well-documented limitation of the classic CASA framework in arid and semi-arid regions^[36]^.

With respect to statistical robustness, our sample size (36 sampling plots) provided sufficient statistical power to detect the effect of site type on carbon sequestration density (power = 83.5%), but had relatively limited power to resolve stand age effects (power = 73.6%). This reduced power is primarily driven by the higher number of stand age groups (six levels) and the uneven distribution of samples across age classes. While we detected a statistically significant effect of stand age (*p* = 0.05), future studies should expand sample sizes or optimize sampling stratification to improve statistical power, enabling more precise quantification of how stand age shapes carbon sequestration density.

Finally, while the allometric biomass models developed from our sampled standard trees exhibited high accuracy at the plot scale, upscaling these relationships to the regional scale requires explicit accounting for spatial heterogeneity in site conditions, stand age, and management practices^[39]^. Future work can further improve the accuracy and robustness of regional estimates in complex terrain by integrating higher-resolution remote sensing data, refining model parameterization via machine learning algorithms, and incorporating long-term continuous monitoring data from fixed field observation stations.

### Prediction of carbon sequestration density year by year

**Figure 11:**
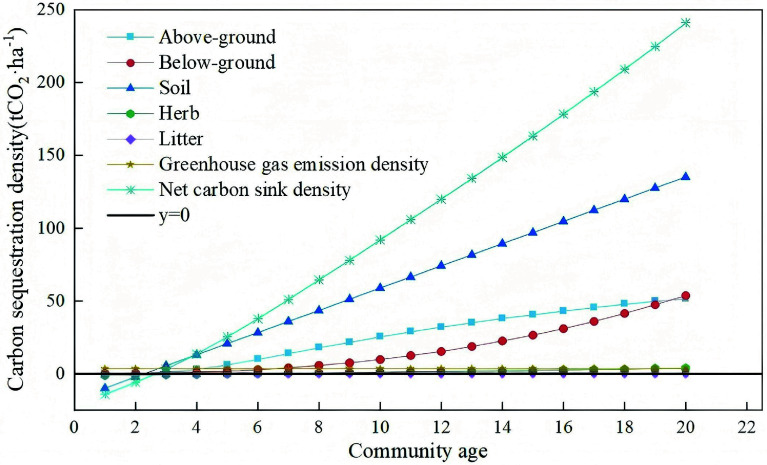
Year-by-year prediction of carbon sequestration density for *Hippophae rhamnoides* L. plantation stands in the study area.

As *Hippophae rhamnoides* L. (sea buckthorn) is a core species for ecological restoration in the Pisha sandstone region, accurate quantification of the carbon sequestration capacity of sea buckthorn plantation stands is critical for regional carbon accounting. Based on field surveys of sea buckthorn stands across a gradient of stand ages, we constructed optimal allometric growth models for above-ground and below-ground biomass, and developed linear models to estimate carbon sequestration density in the herb layer and mineral soil layers as a function of stand age. We then quantified annual cumulative carbon sequestration density for each carbon pool from the first year post-planting, enabling systematic prediction of the carbon sink capacity of sea buckthorn stands across different growth stages (Fig. 11).

The litter layer exhibited unique carbon sequestration dynamics, as its decomposition and accumulation are jointly regulated by microenvironmental conditions and climatic factors. Field measurements revealed substantial interannual variability in litter carbon density with no consistent temporal trend. We therefore adopted the mean value across the monitoring period as a conservative estimate of litter layer carbon sequestration density, to ensure a robust, representative quantification of its contribution to total ecosystem carbon storage.

Notably, initial site preparation and planting activities generated greenhouse gas emissions—predominantly from fossil fuel consumption by machinery—which constitute a form of carbon leakage. These emissions averaged 3.31 tCO_2_ ha^−^^1^ (see Supplementary Table S5) and occurred almost entirely in the first year of afforestation, accounting for approximately 3.6% of the total 10-year carbon sequestration (91 tCO_2_ ha^−^^1^). While these emissions are non-negligible in short-term carbon budgets, the strong carbon uptake capacity of sea buckthorn stands in the mid- and late-growth stages fully offsets these initial emissions over the full plantation life cycle.

Our projection results show that total ecosystem carbon sequestration density increases significantly with stand age, reaching an estimated 91 tCO_2_ ha^−1^ by the end of the 10^th^ growing season, demonstrating substantial carbon sink potential.

In summary, sea buckthorn plantations deliver considerable carbon sink co-benefits in the Pisha sandstone region, with carbon sequestration capacity increasing consistently as stands mature. A net carbon sink is achieved by the third year post-planting. Strategic site selection—prioritizing gully bottoms and shady slopes, for example—can significantly enhance the carbon efficiency of these ecological restoration projects, providing a viable nature-based solution to support regional delivery of China's dual-carbon strategic goals.

### Implications and further research

China's dual-carbon strategic goals—carbon peak by 2030, carbon neutrality by 2060—require maximizing terrestrial ecosystem carbon sequestration potential. Ecosystem restoration and sustainable management are widely proven to significantly enhance terrestrial carbon sinks^[21]^. As an ecologically critical region, the Pisha sandstone region will continue to benefit from policy support for sea buckthorn-based ecological engineering, sustaining its substantial carbon sink potential. Notably, most sea buckthorn plantation stands under these initiatives remain in early growth stages, indicating considerable untapped future sequestration capacity^[40,41]^.

In ecologically fragile regions like the Pisha sandstone area, sea buckthorn-centered restoration projects simultaneously mitigate soil erosion and build substantial carbon sinks, delivering meaningful climate mitigation co-benefits. This provides a valuable model for analogous regions: future ecological engineering should transition from single-objective soil and water conservation to integrated, carbon neutrality-aligned design. Carbon sink enhancement should be embedded into project evaluation frameworks, prioritizing pioneer species (e.g., sea buckthorn) that deliver both ecological resilience and high sequestration potential.

This study also identifies key knowledge gaps for further research. First, soil disturbance and carbon leakage from initial site preparation generate non-negligible short-term emissions. While these were quantified and accounted for here; their long-term impacts on soil properties and sequestration capacity require clarification via continuous *in situ* monitoring. Second, interspecific competition for light, water, and nutrients between sea buckthorn and understory vegetation, and its effects on ecosystem carbon sink structure and stability, remain unresolved critical questions.

The methodology and findings presented here provide a robust scientific basis for assessing sea buckthorn carbon sink function in the Pisha sandstone region, and a transferable framework for nature-based climate solutions in ecologically vulnerable regions across China and globally.

## Conclusions

This study established an integrated framework for assessing carbon sequestration in sea buckthorn plantations by combining multi-temporal remote sensing with systematic field data. The successful application of the CASA model in this arid, complex terrain addresses a key gap in species-specific carbon accounting for fragile ecosystems.

Key findings demonstrate the model's reliability (*R^2^* = 0.79, RMSE = 25.07 tCO_2_·ha^−1^) and reveal that carbon sequestration is spatially heterogeneous, strongest in gully bottoms, and increasing with stand age, with soil as the dominant carbon pool. The established species-specific biomass models showed good transferability to similar habitats, supporting their use for carbon estimation in semi-arid shrublands. Scientifically, this work provides a validated technical pathway for monitoring shrub carbon sinks. Practically, it confirms the significant carbon potential of sea buckthorn restoration, supporting the integration of carbon goals into ecological design—for instance, by prioritizing favorable sites like gully bottoms.

Future research should conduct *in situ* verification and localization of key parameters such as *ε*_max_, incorporate a refined drought stress module based on regional water conditions, and refine parameters with higher-resolution data and investigate long-term dynamics. In summary, sea buckthorn plantations are a proven dual-purpose solution for both soil conservation and quantifiable carbon sequestration, offering a scalable assessment method to inform ecosystem management and climate policy.

## SUPPLEMENTARY DATA

Supplementary data to this article can be found online.

## Data Availability

The datasets generated during and/or analyzed during the current study are available from the corresponding author upon reasonable request.
