# Supplementary material for: Evaluating the CASA model for estimating carbon sequestration in sea buckthorn plantations using multi-temporal remote sensing and field data
Source: For Res (Fayettev). 2026 Apr 9;6:e011. doi: 10.48130/forres-0026-0013 (PMC13195490; doi:10.48130/forres-0026-0013)
Supplement: Supplementary file 1 — Supplementary data to this article can be found online. [file FR-2026-6-0013-S1.zip › 10.48130_forres-0026-0013-Suppl-TableS2.pdf]

Supplementary Table S2 Fitting of *Hippophae rhamnoides* L. multi-growth factor growth model.

	model	a	b	c	d	b_0	R^2	RSS
Ground diameter、chcanopy	$W=aD+bC+b_0$	0.476	0.009			-1.098	0.707	2.07
Ground diameter、plant height	$W=aD+bH+b_0$	0.16	0.908			-0.873	0.821	1.265
plant height、chcanopy	$W=aH+bC+b_0$	0.922	0.005			-0.919	0.825	1.236
Ground diameter、plant height	$a*（D^b）*（H^c）+b_0$	0.248	-0.105	2.694		0.013	0.924	0.54
Ground diameter、plant height	$a*（D^{0.1}）*（H^c）+b_0$	0.228	0.1	2.488		0.006	0.923	0.542
Ground diameter、plant height	$a*（D^{0.5}）*（H^c）+b_0$	0.188	0.5	2.126		-0.001	0.921	0.556
Ground diameter、plant height	$a*（D^2）*（H^c）+b_0$	0.057	2	1.246		0.061	0.897	0.73
Ground diameter、chcanopy	$a*（D^b）*（C^c）+b_0$	2.86E-06	1	2.531		0.038	0.852	1.044
plant height、chcanopy	$a*（H^b）*（C^c）+b_0$	1.65E-01	2.487	0.094		0.004	0.924	0.54
Three growth factors	$W=aD+bH+cC+b_0$	0.092	0.85	0.004		-0.945	0.888	1.218
Three growth factors	$W=aD^b*H^c*C^d+b_0$	0.173	0.1	2.415	0.071	0.002	0.923	0.541

Where D is the ground diameter (cm); H denotes plant height (m);C denoteschcanopy(cm).
