# Supplementary material for: Evaluating the CASA model for estimating carbon sequestration in sea buckthorn plantations using multi-temporal remote sensing and field data
Source: For Res (Fayettev). 2026 Apr 9;6:e011. doi: 10.48130/forres-0026-0013 (PMC13195490; doi:10.48130/forres-0026-0013)
Supplement: Supplementary file 1 — Supplementary data to this article can be found online. [file FR-2026-6-0013-S1.zip › 10.48130_forres-0026-0013-Suppl-TableS3.pdf]

Supplementary Table S3 Fitting of single growth factor growth modelof *Hippophae rhamnoides* L.

	**model**	**a**	**b**	**c**	**d**	**R^2**	**SEE**
ground diameter	$W=a•x+d$	0.204			-0.247	0.551	0.013
	$W=ax+bx^2+d$	-0.561	0.17		0.53	0.83	0.005
	$W=ax+bx^2+cx^3+d$	1.319	-0.671	0.116	-0.768	0.878	0.004
	$W=d•x^a$	1.623			0.037	0.422	0.337
plant height	$W=a•x+d$	0.311			-0.191	0.641	0.011
	$W=ax+bx^2+d$	-0.472	0.281		0.27	0.869	0.004
	$W=ax+bx^2+cx^3+d$	0.934	-0.772	0.233	-0.286	0.892	0.003
	$W=d•x^a$	1.55			0.098	0.562	0.255
chcanopy	$W=a•x+d$	0.006			-0.269	0.516	0.014
	$W=ax+bx^2+d$	-0.016	0		0.54	0.804	0.006
	$W=ax+bx^2+cx^3+d$	0.035	-0.001	0.000002918	-0.645	0.874	0.004
	$W=d•x^a$	1.509			0	0.334	0.388
