# Supplementary material for: Evaluating the CASA model for estimating carbon sequestration in sea buckthorn plantations using multi-temporal remote sensing and field data
Source: For Res (Fayettev). 2026 Apr 9;6:e011. doi: 10.48130/forres-0026-0013 (PMC13195490; doi:10.48130/forres-0026-0013)
Supplement: Supplementary file 1 — Supplementary data to this article can be found online. [file FR-2026-6-0013-S1.zip › 10.48130_forres-0026-0013-Suppl-TableS4.pdf]

Supplementary Table S4 Fitting of *Hippophae rhamnoides* L. multi-growth factor growth model.

	model	a	b	c	d	b_0	R^2	RSS
Ground diameter、chcanopy	$W=aD+bC+b_0$	0.06	0.238			-0.227	0.654	0.246
Ground diameter、plant height	$W=aD+bH+b_0$	0.128	0.003			-0.295	0.589	0.292
plant height、chcanopy	$W=aH+bC+b_0$	0.239	0.002			-0.248	0.662	0.24
Ground diameter、plant height	$a*(D^b)*(H^c)+b_0$	0.059	-2.633	6.624		0.076	0.889	0.079
Ground diameter、plant height	$a*(D^{0.1})*(H^c)+b_0$	0.018	0.1	3.981		0.073	0.885	0.082
Ground diameter、plant height	$a*(D^{0.5})*(H^c)+b_0$	0.015	0.5	3.637		0.073	0.884	0.082
Ground diameter、plant height	$a*(D^2)*(H^c)+b_0$	0.006	2	0.074		0.074	0.881	0.085
Ground diameter、chcanopy	$a*(D^b)*(C^c)+b_0$	3.49E-08	2.872	2.703		2.872	0.879	0.086
plant height、chcanopy	$a*(H^b)*(C^c)+b_0$	9.55E-05	2.983	1.295		0.072	0.888	0.079
Three growth factors	W=aD+bH+cC+b0	0.032	0.214	0.002		-0.257	0.665	0.238
Three growth factors	$W=aD^b*H^c*C^d+b0$	8.91E-05	0.1	2.892	1.299	0.072	0.888	0.08

Where D is the ground diameter (cm); H denotes plant height (m);C denoteschcanopy(cm).
