# Supplementary material for: Evaluating the CASA model for estimating carbon sequestration in sea buckthorn plantations using multi-temporal remote sensing and field data
Source: For Res (Fayettev). 2026 Apr 9;6:e011. doi: 10.48130/forres-0026-0013 (PMC13195490; doi:10.48130/forres-0026-0013)
Supplement: Supplementary file 1 — Supplementary data to this article can be found online. [file FR-2026-6-0013-S1.zip › 10.48130_forres-0026-0013-Suppl-TableS5.pdf]

Supplementary Table S5 Statistical table of CO_2 leakage of the project.

community	Area(km^2)	Seedling transport distance(km)	Water transport distance(km)	Seedling (tCO_2/ha)	land preparation(tCO_2/ha)	Water transport (tCO_2/ha)	Sum(tCO_2/ha)
2013	94.89	15815	569340	0.008	0.150	2.619	2.78
2014	178.69	38716	1072140	0.010	0.217	4.931	5.16
2015	148.83	29766	892980	0.009	0.196	4.107	4.31
2016	135.31	18041	811860	0.006	0.267	3.734	4.01
2017	52.79	5279	316740	0.005	0.139	1.457	1.60
2018	65.23	5436	391380	0.004	0.206	1.800	2.01
mean	112.62	18842.17	675740	0.007	0.196	3.108	3.31
