# Supplementary material for: Evaluating the CASA model for estimating carbon sequestration in sea buckthorn plantations using multi-temporal remote sensing and field data
Source: For Res (Fayettev). 2026 Apr 9;6:e011. doi: 10.48130/forres-0026-0013 (PMC13195490; doi:10.48130/forres-0026-0013)
Supplement: Supplementary file 1 — Supplementary data to this article can be found online. [file FR-2026-6-0013-S1.zip › 10.48130_forres-0026-0013-Suppl-TableS1.pdf]

Supplementary Table S1 Fitting of single growth factor growth model of *Hippophae rhamnoides* L.

	model	a	b	c	d	R^2	SEE
ground diameter	$W=a\bullet x+d$	0.709			-0.949	0.67	0.097
	$W=ax+bx^2+d$	-1.125	0.407		0.912	0.832	0.05
	$W=ax+bx^2+cx^3+d$	3.139	-1.499	0.263	-2.029	0.857	0.042
	$W=d\bullet x^a$	2.735			0.045	0.712	0.282
plant height	$W=a\bullet x+d$	1.102			-0.775	0.812	0.055
	$W=ax+bx^2+d$	-0.628	0.62		0.243	0.846	0.022
	$W=ax+bx^2+cx^3+d$	-0.503	0.526	0.021	0.194	0.871	0.022
	$W=d\bullet x^a$	2.427			0.234	0.82	0.176
chcanopy	$W=a\bullet x+d$	0.02			-1	0.605	0.116
	$W=ax+bx^2+d$	-0.035	0		1.001	0.783	0.064
	$W=ax+bx^2+cx^3+d$	0.079	-0.001	0.0000064	-1.602	0.817	0.054
	$W=d\bullet x^a$	2.545			0.000005	0.565	0.426
